# Sex differences in adult social, cognitive, and affective behavioral deficits following neonatal phlebotomy‐induced anemia in mice

**DOI:** 10.1002/brb3.1780

**Published:** 2021-02-19

**Authors:** Tatyana M. Matveeva, Garima Singh, Tate A. Gisslen, Jonathan C. Gewirtz, Michael K. Georgieff

**Affiliations:** ^1^ Department of Psychology University of Minnesota Minneapolis MN USA; ^2^ Department of Pediatrics University of Minnesota Minneapolis MN USA; ^3^ Department of Neuroscience University of Minnesota Minneapolis MN USA

**Keywords:** anemia, animal models, behavioral deficits, phlebotomy, prematurity, sex differences

## Abstract

**Introduction:**

Anemia is common in prematurely born infants due to blood loss resulting from frequent phlebotomies and may contribute to their neurobehavioral deficits. Preclinical models of phlebotomy‐induced anemia (PIA) have revealed metabolic and genomic changes in multiple brain structures of young mice, yet the impact of neonatal PIA on early‐life and adult behavior has not been assessed.

**Methods:**

The present study employed a range of behavioral measures in phlebotomized anemic neonatal mice to investigate short‐ and long‐term neurodevelopmental effects. PIA from postnatal (P) days 3 to 14 caused sex‐specific changes in social behavior, novelty preference, and anxiety at P17 that persisted into adulthood.

**Results:**

Our preclinical model suggests that PIA may contribute to acute and long‐term behavioral and affective deficits and warrants further substantiation of the observed behavioral phenomena in larger samples.

**Conclusions:**

We conclude that this model is a useful tool for beginning to better understand the lasting effect that early‐life PIA might have on the developing brain. The differential impact of PIA on male and female subjects warrants further exploration for the development of appropriately targeted interventions.

## Graphical abstract

The impact of neonatal PIA on early‐life and adult behavior has not been assessed. The present study employed a range of behavioral measures in phlebotomized anemic neonatal mice to investigate short‐ and long‐term neurodevelopmental effects. PIA from postnatal (P) days 3 to 14 caused sex‐specific changes in social behavior, novelty preference, and anxiety at P17 that persisted into adulthood. The differential impact of PIA on male and female subjects warrants further exploration for the development of appropriately targeted interventions.

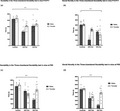

## 1 INTRODUCTION

Premature births make up approximately 10% of all live births worldwide each year (WHO, 2016). Children born prematurely are at increased risk of behavioral, cognitive, and affective deficits both early and later in life, show higher incidence of academic underachievement, and have impairments in attention, emotional regulation, and executive function (Aarnaoudse‐Moens, Smidts, Duivenvoorden, & Weisglas‐Kuperus, 2009; van Baar, Vermaas, Knots, de Kline, & Soons, 2009; Delobel‐Ayoub et al., 2009; Doyle & Anderson, 2010; Lukowski et al., 2010).

While multiple factors contribute to this increased neurodevelopmental risk, the degree of neonatal anemia is one that is controllable by the healthcare team. However, two randomized trials that assessed neurodevelopmental outcomes as a function of the degree of neonatal anemia were unable to determine the degree of anemia that is safe for the developing brain. The Iowa trial (Bell, 2005) showed increased risk of severe hemorrhage and the PINT trial (Kirpalani et al., 2006) showed poorer overall performance on standardized developmental testing in more anemic infants. Long‐term follow‐up of infants from the Iowa trial demonstrated poorer long‐term outcome only in males, whereas females with lower hematocrits had better outcomes than those randomized to higher hematocrits (McCoy et al., 2011; Nopoulos et al., 2011). Transfusion is a potential risk factor to the developing organs, including the brain (Benavides et al., 2019). Because of the apparent risks of transfusions, fewer infants are being transfused and more infants are allowed to maintain lower hematocrits without consideration of potential neurometabolic (Wallin et al., 2015, 2017) and behavioral consequences.

Anemia is a risk to the developing brain because it compromises the delivery of key metabolic and energetic substrates, such as iron and oxygen. In preterm infants, much of anemia is caused by significant phlebotomy‐induced blood loss (Carroll & Widness, 2012; Widness, 2008). Studies indicate that inadequate iron and oxygen status early in life has a substantial negative effect on the development of the brain as well as other organ systems (Lozoff & Georgieff, 2006). In particular, early‐life iron deficiency is predictive of subsequent mental health complications, including severe psychopathology (Barks, Fretham, Georgieff, & Tran, 2018; Insel, Schaefer, McKeague, Susser, & Brown, 2008; Lozoff & Georgieff, 2006; Schmidt, Tancredi, Krakowiak, Hansen, & Ozonoff, 2014).

Preclinical studies show that phlebotomy‐induced anemia reduces brain iron concentrations by 40% (Wallin et al., 2015). Iron deficiency itself, regardless of systemic anemia, is critical for the progression of these deficits (Barks et al., 2018; Carlson et al., 2010; Fretham, Carlson, & Georgieff, 2011, 2013; Pisansky et al., 2013; Wallin et al., 2015, 2017). Changes in cerebral energy metabolism, neurotransmitter metabolism, myelination, and dendritic structure secondary to early‐life iron deficiency anemia have been shown in rodent models (Carlson et al., 2009; Rao, Tkac, Townsend, Gruetter, & Georgieff, 2003; Ward et al., 2007). In humans, prenatal and neonatal iron deficiency have been linked to abnormalities in memory (Geng et al., 2015; Siddappa et al., 2004), temperament (Wachs, Pollitt, Cueto, Jacoby, & Creed‐Kanashiro, 2005), psychomotor function (Tamura et al., 2002), and auditory system neural myelination (Amin, Orlando, & Wang, 2013). Infants with early‐life iron deficiency anemia exhibit greater anxiety or stress in the presence of unfamiliarity (Angulo‐Kinzler, Peirano, Lin, Algarin, et al., 2002; Angulo‐Kinzler, Peirano, Lin, Garrido, & Lozoff, 2002; Lozoff, Jimenez, Hagen, Mollen, & Wolf, 2000), reduced social interaction and ability to be soothed, and greater negative emotionality and passivity (Lozoff et al., 2003). Several studies have indicated lower academic achievement measured by performance in specific domains (i.e., reading, arithmetic, spelling, problem solving) and a progressive increase in the severity of these deficits during development (Espy, Fang, Charak, Minich, & Taylor, 2009; Hagen, Palta, Albanese, & Sadek‐Badawi, 2006). Additional research suggests that these effects differentially affect male and female preterm children (Saigal, Hoult, Streiner, Stoksopf, & Rosenbaum, 2000; Whitfielf, Grunau, & Holsti, 1997). Sex differences in outcomes of anemic preterm children have also been shown (Benavides et al., 2019; McCoy et al., 2011; Nopoulos et al., 2011). Although their significance is clear, the etiology of such long‐term, sex‐specific cognitive and behavioral deficits remains poorly understood because of the multiple confounding variables inherent to these clinical populations. Preclinical models investigating sex‐specific structural and functional effects of early‐life anemia can provide insights into the mechanisms of cognitive and behavioral changes in preterm infants (Benavides et al., 2019; Nopoulos et al., 2011).

The objective of the present study was to examine the acute and long‐term behavioral effects of early‐life phlebotomy‐induced anemia (PIA) in an established mouse model (Wallin et al., 2015, 2017). In that model, PIA with a hematocrit of 25% increased hippocampal lactate concentrations by 60% and reduced expression of critical synaptic plasticity genes, for example, BDNF, in the neonatal period (Wallin et al., 2015, 2017). In this study, we employed a set of behavioral paradigms with the same model to assess a broad range of functional domains (social behavior, anxiety, cognitive performance) in phlebotomized male and female mice at postnatal (P) day 17 and again in adulthood starting at P65. Further, we tested the effects of two postphlebotomy hematocrit (Hct) target levels (25% Hct and 18% Hct) on these measures to establish whether a dose‐dependent effect was present. The chosen Hct values were selected based on human data (Bell, 2005; Kirpalani et al., 2006), using the same percent reduction relative to control (nonphlebotomized) hematocrits utilized in the human studies. Specifically, while physiologic anemia and neonatal Hct in mice are similar to those observed in humans, the mice used in the present model had a lower starting Hct (38%) than values recorded in the human (50%). Thus, in addition to achieving 25% Hct following phlebotomy, a second target Hct level of 18% was included to reflect the same percent separation from nonphlebotomized controls as is present in humans. The study design also allowed for dose–response analysis as a function of degree of anemia.

## 2 METHODS

### 2.1 Animal preparation

The study was conducted with the approval of the Institutional Animal Care and Use Committee at the University of Minnesota. Wild‐type C57/BL6 animals were used for all the experiments in the study. Pregnant and lactating dams were fed standard chow containing ~200 ppm of iron (Envigo; Indianapolis, IN) and were given access to food and water ad libitum. The animals were maintained on a 14‐hr light/10‐hr dark cycle with humidity and environmental temperature controlled as per the institution's Research Animal Resources guidelines. Litters were culled to 8–9 pups at P2 or, in the case of smaller litters, foster pups were added to maintain similar litter size. This was done to reduce variability of food access and growth rates resulting in disproportionate weight gain. The pups along with the lactating dams were transferred to the behavioral testing facility at P14 and were allowed to acclimatize to their new environment for 2–3 days before the beginning of testing. Mice tested at postnatal day 17 will be referred to as “P17” mice, followed by their Hct status in the remainder of the study, while mice tested after postnatal day 65 will be referred to as “P65” mice, followed by prior Hct status. A breakdown of the number of animals of each sex included in each of the treatment groups is provided separately for every task below.

### 2.2 Phlebotomy‐induced anemia

Neonatal mice were phlebotomized from postnatal (P) day 3 to P13 via facial vein venipuncture using a micropipette as described previously (Wallin et al., 2015, 2017). Pups were weighed daily to determine the quantity of blood to be drawn. For both 25% hematocrit and 18% hematocrit groups, blood was drawn twice daily at 5.25 µL/g until goal hematocrit levels were reached (P7‐P8 for the 25% group and P10‐P11 for the 18% group). The animals were bled daily at 3.5 µl/g thereafter to maintain the target hematocrit levels. The hematocrit was measured daily by centrifugation of microhematocrit collection tubes at 10,000 rpm for 5 min and quantified using a hematocrit card reader. Control pups received a nonphlebotomizing needle prick to the nape of the neck on the same schedule as phlebotomized pups. They were handled similarly, including time spent away from the dams, to the phlebotomized pups in order to minimize variability in stress responses. Each litter comprised pups from both control and phlebotomized groups.

### 2.3 Three‐chambered social approach task

We tested 19 nonbled controls (males = 8), 21 25% Hct mice (males = 7), and 19 18% Hct mice (males = 9) at P17, and 13 controls (males = 5), 22 25% Hct mice (males = 8), and 21 18% Hct mice (males = 9) at P65. The three‐chambered social approach task was used to assess sociability and social approach behavior^25^. In this experiment, animals underwent three 10‐min phases within an arena (20 × 25 × 45 cm) partitioned into three chambers. In the first (habituation) phase, individual mice were allowed to explore the arena freely. In the second (sociability) phase, an age‐ and sex‐matched conspecific was enclosed under a small wire mesh cage (cup) within one chamber (hereafter referred to as the “mouse” chamber), while the other chamber contained an empty cup. The side of the apparatus containing the conspecific was randomized. In the third phase of the task, a novel conspecific was enclosed under a wire mesh cup on the opposite side of the chamber. For each phase, the time spent within each chamber and investigating each wire cage were scored by a genotype‐naïve (blind) experimenter. The ratio of time spent in the novel versus familiar chamber was calculated during the novelty phase.

### 2.4 Novel object recognition (NOR)

NOR test procedures were similar to those described previously by our laboratory^26^ and others^27^. A total of 19 nonbled controls (males = 12), 21 25% Hct mice (males = 13), and 20 18% Hct mice (males = 9) were tested in this task. Both acquisition and test phases were videorecorded. Prior to each phase, animals were habituated to the test chamber for 1 min. Mice were then removed, objects were placed in the chamber, and the animals were returned to the chamber. During the acquisition phase, two identical objects were placed approximately 5 cm away from the opposite walls of the chamber and mice were allowed to explore freely for 5 min. At the end of the trial, mice were removed and then returned to the chamber for the test phase 15 min later. During the test phase, the familiar object from the acquisition phase was placed in one location within the chamber and a novel object was placed in the opposite location. Animals were placed in the test chamber containing both objects for five minutes. Upon completion of the test phase, mice were returned to their home cages. The test chamber was thoroughly wiped down with 70% alcohol, and objects were cleaned with diluted bleach between sessions.

The test phase made use of three different pairs of novel and familiar objects (for a total of 6 objects), which varied in color, material, texture, and shape. The location of the novel object in the test chamber, as well as test order and object pair, was counterbalanced across conditions. Object investigation during testing was scored by trained group‐naive raters. During the acquisition phase of the task, time spent exploring either of the two identical objects placed in the testing apparatus was recorded. The total duration of investigation was calculated for each mouse (exploration score). During the test phase, a normalized novelty score was calculated for each animal by dividing the difference in investigation times for the novel and familiar objects by the time spent investigating the familiar object. Preference for the novel object (and memory for the familiar object) was defined as time spent investigating the unfamiliar object compared to chance during the test phase.

### 2.5 Elevated plus maze

19 nonphlebotomized controls (males = 8), 22 25% Hct mice (males = 8), and 22 18% Hct mice (males = 10) aged P65 were tested in this task. Testing was performed as described elsewhere (Carola et al., 2002). The apparatus consisted of two open arms (30 × 5 cm) and two closed arms (30 × 5 cm, surrounded by 10 cm high walls). Each pair of identical arms were placed opposite of each other and converged at a central square platform (5 × 5 cm). The base of the arms and central platform was made of gray Plexiglass, while the walls of the closed arms were made of black Plexiglass. The apparatus was elevated 45 cm above the floor and lit by four red‐light lamps (4 × 60 W) placed above each arm. The animals were tested during the first half of the dark phase of their light/dark cycle. The test was initiated by placing the mouse on the central platform of the maze, facing one of the open arms, and letting it move freely. Each session lasted 5 min. Mouse behavior was videotaped by a video camera placed above the apparatus. Test session videotapes were scored using AnyMaze (Stoelting Co., Wood Dale, IL, USA).

### 2.6 Open field test

Data from 18 nonbled controls (males = 7), 21 25% Hct mice (males = 7), and 22 18% Hct mice (males = 10) were obtained in this task. Exploration of an open field was used to measure anxiety‐like behavior. Animals were placed in an open field chamber (50 × 50 cm), and activity was recorded by a digital camera for 10 min. The amount of time mice engaged in thigmotaxis, as well as time spent in the center and center‐adjacent areas of the chamber, was assessed using AnyMaze (Stelting Co., Wood Dale, IL, USA).

### 2.7 Statistical analysis

Data were analyzed using GraphPad Prism. To assess the effect of sex and treatment on behavior, we used 2‐way ANOVA and performed Tukey's and Sidak's multiple comparisons tests.

## 3 RESULTS

### 3.1 Three‐chambered social approach task

**FIGURE 1:**
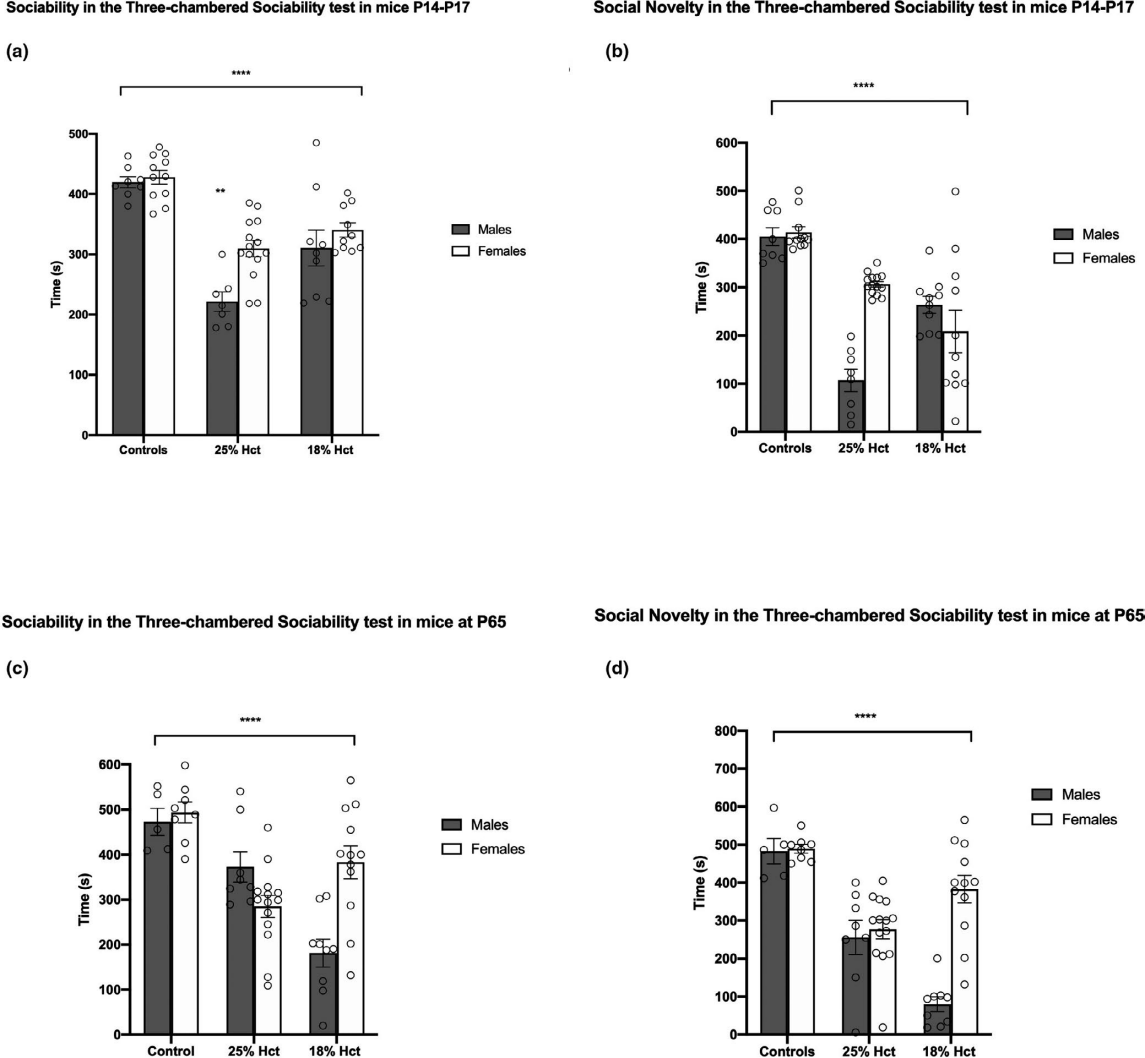
Sociability and social novelty behavioral data in the three‐chambered social approach task at P17 and P65. Panel a: 2‐way ANOVA of sociability behavior in P17 mice (19 nonbled controls (males = 8), 21 25% Hct mice (males = 7), and 19 18% Hct mice (males = 9)). Asterisks show main effects of treatment (*p* < .0001) and sex (*p* < .01). Panel b: 2‐way ANOVA of behavior in the social novelty phase for P17 mice. Asterisks show main effects of treatment (*p* < .0001). There was a main effect of sex (*p* < .05) and a sex × treatment interaction (*p* < .0001). Panel c: 2‐way ANOVA of sociability behavior in P65 mice (13 nonbled controls (males = 5), 22 25% Hct mice (males = 8), and 21 18% Hct mice (males = 9)). Asterisks denote the main effect of treatment (*F*(2, 50) = 17.38, *p* < .0001); there was a significant sex × treatment interaction (*F*(2, 50) = 11.77, *p* < .0001). Panel d: 2‐way ANOVA of behavior in the social novelty phase for P65 mice. There was a main effects of sex (*F*(1, 50) = 16.49, *p* = .0002), a main effect of treatment (*F*(2, 50) = 29.34, *p* < .0001), and a significant sex × treatment interaction (*F*(2, 50) = 14.10, *p* < .0001) (denoted by ****). Simple effects not shown with ***. *p < .05; **p < .01; ***p < .001; ****p < .0001. Means ± *SEM*

To test whether sex and treatment (hematocrit status) had an impact on behavior in this task and to examine the possible interaction between the two, we performed a 2‐way ANOVA, with sex and treatment as factors (Figure 1). Among P17 animals, both treatment (*F*(2, 53) = 44.65, *p* < .0001) and sex (*F*(1, 53) = 9.340, *p* = .0035) were related to significant differences in sociability, although there was no significant interaction between the two (*F*(2, 53) = 2.983, *p* = .0592), possibly due to small sample sizes. Post hoc tests revealed that phlebotomized male and female mice demonstrated reduced sociability relative to nonbled controls and that 25% Hct males showed the greatest impairment in sociability (*M* = 221.571, *SD* = 42.54, *p* < 0001 relative to male controls, and *p* = .0033 relative to 18% Hct males) (Figure 1a). Similarly, phlebotomized mice spent less time investigating a novel conspecific in the social novelty portion of the experiment relative to controls. Analyses revealed a main effect of sex (*F*(1, 56) = 6.904, *p* = .0111) and treatment (*F*(2, 56) = 41.19, *p* < .0001) and a significant sex × treatment interaction (*F*(2, 56) = 16.06, *p* < .0001. Figure 1b). Once again, 25% Hct males showed the greatest deficit in social novelty relative to controls (*M* = 106.875, *SD* = 65.8, *p < *.0001) and compared to 18% Hct males (*M* = 106.875, *SD* = 65.8, *p* = .0001) (Figure 1b).

**FIGURE 2:**
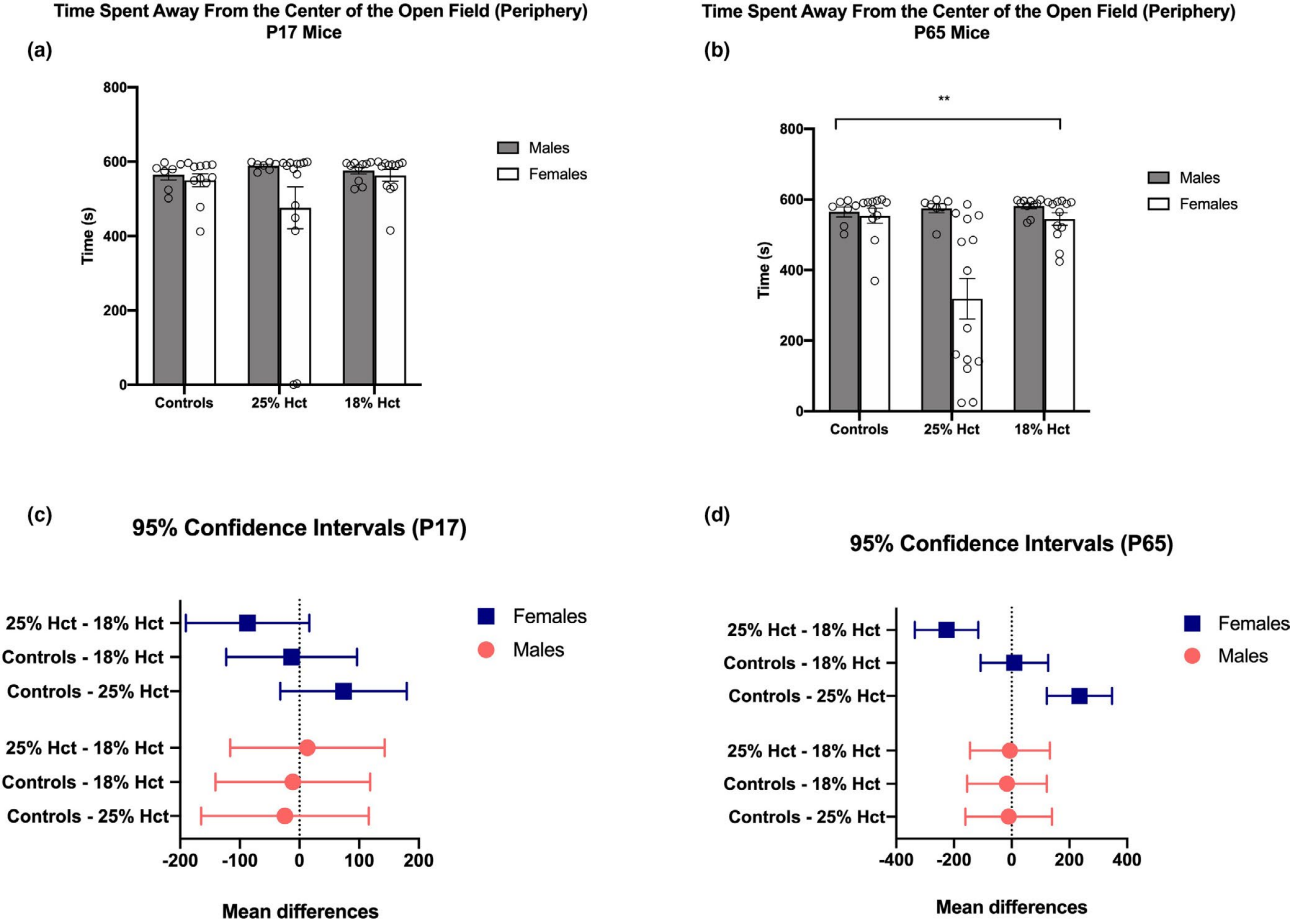
Confidence intervals based on post hoc tests of P17 and P65 three‐chambered sociability test data. Panels a,b: sociability (a) and social novelty (b) in P17 mice. Panels c‐d: sociability (c) and social novelty (d) in P65 mice

At P65, phlebotomized mice showed reduced sociability relative to controls. Analyses revealed no effect of sex (*F*(1, 50) = 2.773, *p* = .1021), a main effect of treatment (*F*(2, 50) = 17.38, *p* < .0001), and a significant treatment × sex interaction (*F*(2, 50) = 11.77, *p* < .0001). Notably, the impairment in sociability was greatest in 18% Hct males (Figure 1c). In the social novelty phase of the task, control mice performed significantly better than both phlebotomized groups (Figure 1d). There was a main effects of sex (*F*(1, 50) = 16.49, *p* = .0002), a main effect of treatment (*F*(2, 50) = 29.34, *p* < .0001), and a significant sex × treatment interaction (*F*(2, 50) = 14.10, *p* < .0001). 18% Hct males demonstrated the most pronounced deficit in social novelty relative to all other groups (*M* = 79.89, *SD* = 57.24 *p < *.0001 compared to controls males, *p* = .0066 compared to 25% Hct males) (Figure 1d). Importantly, while 25% Hct females exhibited reduced sociability to control females (*M* = 277.05, *SD* = 95.19*, p* = .0001), neither 18% Hct females and controls female mice nor 18% Hct and 25% Hct females differed (Figure 1d). Confidence intervals and mean differences are depicted in Figure 2a–d.

### 3.2 Open field

**FIGURE 3:**
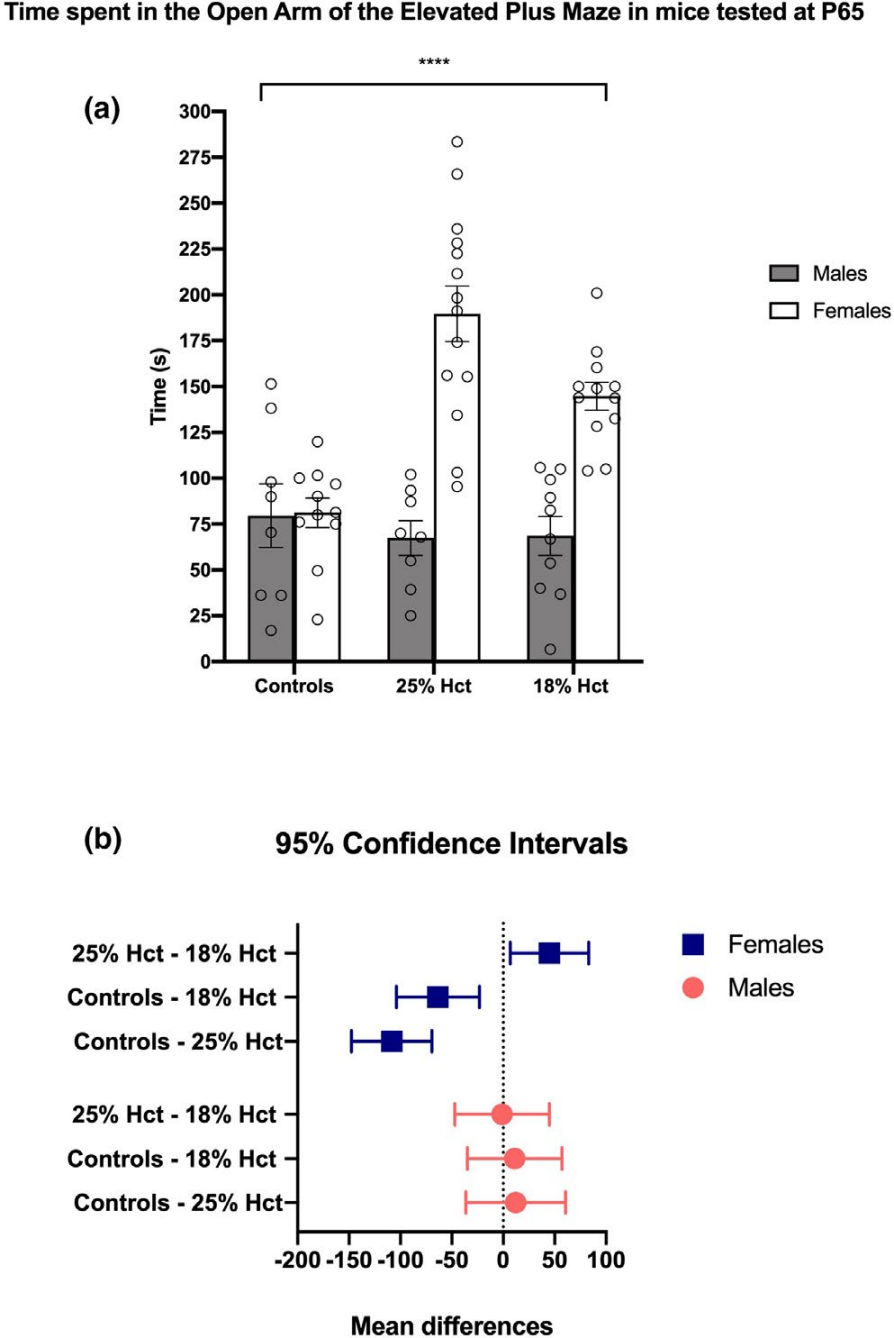
P17 and P65 performance in the open field test. Subjects: 18 nonbled controls (males = 7), 21 25% Hct mice (males = 7), and 22 18% Hct mice (males = 10). Panel a: 2‐way ANOVA of P17 open field behavior. No differences between treatments were found. Panel b: 2‐way ANOVA of P65 open field behavior. Asterisks denote a main effect of sex (*F*(1, 55) = 11.28, *p* = .0014), treatment (*F*(2, 55) = 6.366, *p* = .033), and a significant sex × treatment interaction (*F*(2, 55) = 6.549, *p* = .0028). Tukey HSD analyses showed that 25% Hct female mice spent significantly less time in the periphery of the open field (*M* = 318.807, *SD* = 215.361, *p* < 000.1). Panels c–d: confidence intervals based on post hoc tests of P17 and P65 data, respectively. “Mean differences” (x‐axis) reflects the differences between means. *p < .05; **p < .01; ***p < .001; ****p < .0001. Means ± *SEM*

The three groups did not differ in performance in the open field test at P17. No effect of sex (*F*(1, 55) = 2.629, *p* = .1107) or treatment (*F*(2, 55) = 0.5861, *p* = .5599) was observed (Figure 3a). However, a main effect of sex (*F*(1, 55) = 11.28, *p* = .0014), treatment (*F*(2, 55) = 6.366, *p* = .033), and a significant sex × treatment interaction (*F*(2, 55) = 6.549, *p* = .0028) emerged at P65. Tukey HSD analyses demonstrated no differences between control and 18% Hct animals regardless of sex, and significantly less time spent in the periphery of the open field by 25% Hct female mice (*M* = 318.807, *SD* = 215.361, *p* < 000.1) (Figure 3b). Confidence intervals and mean differences are depicted in Figure 3c–d.

### 3.3 Elevated plus maze

**FIGURE 4:**
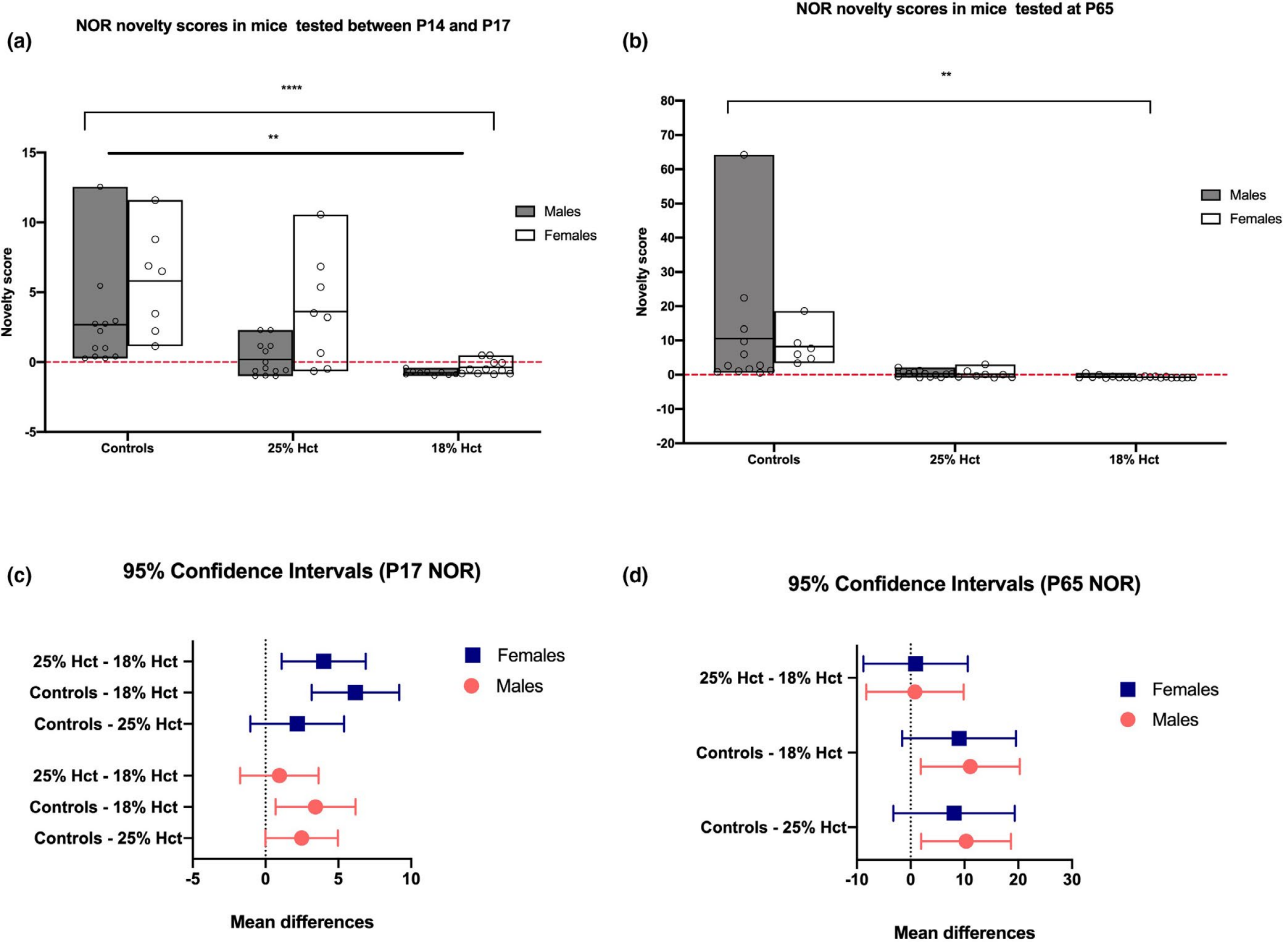
P65 elevated plus maze behavioral data from 19 nonphlebotomized controls (males = 8), 22 25% Hct mice (males = 8), and 22 18% Hct mice (males = 10). Panel a: A 2‐way ANOVA revealed a main effect of sex (*F*(1, 57) = 43.18, *p* < .0001) and treatment (*F*(2, 57) = 7.239, *p* = .0016), and a significant sex × treatment interaction (*F*(2, 57) = 11.50, *p* < .0001), denoted by ****. Panel b: confidence intervals based on post hoc tests of P65 data. “Mean differences” (x‐axis) reflects the differences between means. *p < .05; **p < .01; ***p < .001; ****p < .0001. Means ± *SEM*

All animals tested in this task were adult (P65). A 2‐way ANOVA revealed a main effect of sex (*F*(1, 57) = 43.18, *p* < .0001) and treatment (*F*(2, 57) = 7.239, *p* = .0016), and a significant sex × treatment interaction (*F*(2, 57) = 11.50, *p* < .0001). Post hoc analyses divulged no differences in task performance among male mice of all 3 groups. However, both 25% Hct female mice (*M* = 189.7, *SD* = 56.72, *p* < .0001) and 18% Hct females (*M* = 144.75, *SD* = 26.48, *p = *.0009) spent more time in the open arm of the EPM than did control females (*M* = 81.25, *SD* = 26.55). Additionally, 18% Hct female animals spent more time in the open arm of the maze than did 25% Hct females (*M* = 144.75, *SD* = 26.48, *p = *.0159) (Figure 4). Confidence intervals and mean differences are depicted in Figure 4b.

### 3.4 Novel object recognition

**FIGURE 5:**
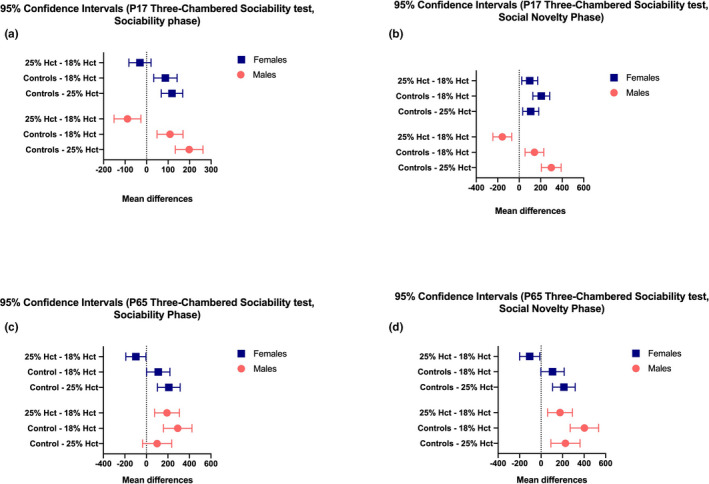
P17 and P65 NOR novelty scores (time(novel‐time(familiar)/time(familiar) from 19 nonbled controls (males = 12), 21 25% Hct mice (males = 13), and 20 18% Hct mice (males = 9). Panel a: 2‐way ANOVA revealed main effect of sex (*F*(1, 54) = 12.09, *p* = .0010, denoted by **), a main effect of treatment (*F*(2, 54) = 16.98, *p* < .0001, denoted by ****) but no sex × treatment interaction. Of 18% Hct males had significantly lower novelty scores than male controls (*M* = −0.76, *SD* = 0.175, *p* = .0095, Tukey HSD). Of 18% female mice had lower novelty scores than controls (*M* = −0.37, *SD* = 0. 512, *p* < .0001, Tukey HSD), and the novelty scores of 18% Hct females were significantly lower than those of 25% Hct females (*M* = −0.37, *SD* = 0. 512, *p* = .0037, Tukey HSD). Panel b: 2‐way ANOVA revealed a main effect of treatment (*F*(2, 53) = 7.403, *p* = .0015, denoted by **) but not sex (*F*(1, 53) = 0.1351, *p* = .7146), and no significant sex × treatment interaction (*F*(2, 53) = 0.0933, *p* = .9111). 25% Hct (*M* = 0.2201, *SD* = 0.998, *p* = .0060, Tukey HSD) and 18% mice (*M* = −0.6403, *SD* = 0.343, *p* = .0025, Tukey HSD) had significantly lower novelty scores compared to controls. Phlebotomized mice did not differ from each other. Panels c–d: confidence intervals based on post hoc tests of P17 and P65 data, respectively. “Mean differences” (x‐axis) reflects the differences between means. *p < .05; **p < .01; ***p < .001; ****p < .0001

Preference for the novel or familiar object was measured as time spent exploring (sniffing, touching, climbing on) and facing the object. A novelty score was calculated for each animal in each group by dividing the difference between time spent investigating novel object and time investigating the familiar object divided by the time spent investigating the novel object [time(novel)‐time(familiar)/time(familiar)]. At P17, phlebotomized mice exhibited behaviors consistent with novelty aversion as evidenced by lower novelty scores. Analyses revealed a main effect of sex (*F*(1, 54) = 12.09, *p* = .0010), a main effect of treatment (*F*(2, 54) = 16.98, *p* < .0001) but no sex × treatment interaction. Post hoc analyses showed no differences between control and 25% Hct males, and between males from the two phlebotomized groups. However, 18% Hct males had significantly lower novelty scores than male controls (*M* = −0.76, *SD* = 0.175, *p* = .0095). 25% Hct females also did not differ from control females. In contrast, 18% female mice had lower novelty scores than controls (*M* = −0.37, *SD* = 0. 512, *p* < .0001). Similarly, 18% Hct females' novelty scores were significantly lower than those of females in the 25% Hct group (*M* = −0.37, *SD* = 0. 512, *p* = .0037) (Figure 5a).

At P65, sex did not contribute to differences in task performance across treatments (*F*(1, 53) = 0.1351, *p* = .7146). There was a main effect of treatment (*F*(2, 53) = 7.403, *p* = .0015) but no sex × treatment interaction (*F*(2, 53) = 0.0933, *p* = .9111). Tukey's HSD tests revealed that both 25% Hct (*M* = 0.2201, *SD* = 0.998, *p* = .0060) and 18% mice (*M* = −0.6403, *SD* = 0.343, *p* = .0025) had significantly lower novelty scores compared to controls. No differences existed between 25% Hct and 18% Hct mice (Figure 5b). Confidence intervals and mean differences are depicted in Figure 5c–d.

## 4 DISCUSSION

Whether anemia without the neurodevelopmental risk of red cell transfusion (Benavides et al., 2019) is sufficient to compromise neurodevelopment, and whether deficits associated with anemia are short‐lived or lasting, is poorly understood. Here, we addressed these questions in a validated preclinical model of phlebotomy‐induced neonatal anemia (Wallin et al., 2015, 2017). Two Hct target concentrations (25% and 18%) allowed us to test the effect of PIA on cognitive, social, and affective functioning in a dose‐dependent manner. These concentrations were chosen to be commensurate with those seen in human preterm infants after adjusting for lower starting Hcts in the mouse. We examined whether PIA produced acute effects on behavior and whether observed differences persisted into adulthood. We also assessed whether PIA produced different acute and long‐term effects on males and females.

Previous studies using this neonatal PIA model have found evidence for brain hypoxia, as indicated by elevated VEGF expression in the hippocampus. Additionally, these studies have revealed a 40% reduction in total brain iron and an increase in hippocampal transferrin receptor‐1 expression, confirming the presence of brain iron deficiency (Wallin et al., 2015, 2017). This model is also characterized by brain acidosis, evidenced by a 60% increase in hippocampal lactate concentration, and altered expression of hippocampal synaptic plasticity genes, including BDNF. Consistent with these findings of physiological dysregulation, in this study we found evidence consistent with impaired hippocampal function in the performance of PIA animals on the NOR task. The performance of PIA mice on this task was related to early Hct status, in that more severely anemic 18% Hct mice spent less time investigating the novel object, while 25% Hct mice showed a more moderate impairment. The observed deficits in novelty preference persisted into adulthood despite resolution of PIA, suggesting that anemia‐induced changes to hippocampal integrity in early development have a lifelong effect on behavioral outcomes and that the severity of these outcomes is related to severity of anemia. Given the aforementioned tendency to decrease the number of transfusions in cases of neonatal anemia in NICUs, our findings highlight the possible long‐term risks of prolonged exposure to increasingly severe anemia in preterm infants.

Beyond neurocognitive deficits, growing evidence demonstrates that preterm infants are at a markedly elevated risk for developing sociocognitive abnormalities including autism spectrum disorder (ASD) (Agarwal et al., 2018; Johnson et al., 2010; Limperopoulos et al., 2008; Mahoney, Minter, Burch, & Stapel‐Wax, 2013). Although the neural mechanisms implicated in the etiology of sociocommunicative deficits and ASD remain incompletely understood, known risk factors include male gender, lower birthweight, gestational age, and infection (Limperopoulos et al., 2008; Ng, de Montigny, Ofner, & Do, 2017). Our study newly implicates neonatal anemia as another risk factor in the development of aberrant social behavior. In the PIA model, this included aversion to novel conspecifics and preference for nonsocial environments. An impairment in sociability and social novelty preference in the three‐chambered social approach task was shown suggested a relationship between neonatal anemia and neurobehavioral deficits. Our results reveal that early (P17‐P21) changes in social behavior impact males and females in both phlebotomized groups, though they were most pronounced in 25% Hct males, and that abnormalities persisted into adulthood. Both sociability and social novelty were deficient in 25% and 18% Hct animals at P17‐P21 and did not reach control levels at P65. Notably, at P65, 25% Hct males performed better than 18% Hct males in the sociability portion of the task, and the latter spent the least amount of time in the compartment containing a conspecific versus the empty compartment. Similarly, 18% Hct males (P65) exhibited strong social novelty aversion; indeed, we observed a graded effect of treatment on performance in the social novelty phase of the experiment among male mice. Our results suggest a stronger impact of treatment in males on performance in the three‐chambered sociability test, a result consistent with findings in the clinical literature.

Iron deficiency causes substantial changes in monoamine metabolism throughout the brain, with particularly large effects on the dopamine system (Beard, 2003; Chen, Beard, & Jones, 1995; Lozoff, 2011; Lozoff & Georgieff, 2006; Unger et al., 2012; Yehuda & Youdim, 1989; Youdim & Green, 1978). Such changes may explain iron‐deficient infants’ heightened risk for anxiety, as indicated by increased fear, wariness, and hesitancy (for a detailed review, see Lozoff, 2011). Previous preclinical studies utilizing the open field test to measure anxiety‐associated behaviors in iron‐deficient rats have reported subtle changes in indexes of arousal, such as defecation, as well as diminished rearing behaviors (Felt et al., 2006; Felt & Lozoff, 1996). Others have reported a relationship between iron deficiency and elevated anxiety‐like behaviors in the EPM (Li et al., 2011), although the literature linking iron deficiency and anxiety offers mixed findings (Beard, Erikson, & Jones, 2002; Eseh & Zimmerberg, 2005; Gewirtz, Hamilton, Babu, Wobken, & Georgieff, 2008). Our current findings indicate that neonatal anemia results in altered affect, as assessed in the open field and EPM tasks, and that this phenotype disproportionately affects females. Specifically, while no differences were observed early in life, we found that 25% Hct female adult animals spent more time than controls in the center of the open field. Furthermore, P65 female mice spent more time in the open arms of the EPM and had shorter stays in the closed arms, while males of either phlebotomized group did not differ from control animals.

Preclinical models of neonatal anemia due to dietary iron deficiency result in acute neurodevelopmental and behavioral morbidity (Beard et al., 2006; Pinero, Li, Connor, & Beard, 2000; Ward et al., 2007). In light of this, the evidence presented here for compromised cognitive, social, and affective functioning early during development was expected. More surprising, however, was the enduring nature of these effects. The persistence of deficits into adulthood across several domains suggests that the functional integrity of multiple brain structures is not fully restored despite iron repletion. Such effects are concerning from a clinical standpoint in view of our limited understanding of the sustained effects of neonatal anemia on the developing brain and the potential societal costs of lifelong morbidity.

Two compatible theoretical explanations can account for the persistence of these behavioral changes. According to the critical period theory, regional brain structures undergo development during a critical period, disruption of which results in lasting structural and associated functional changes. Such effects have been observed as a result of fetal/neonatal iron deficiency and iron deficiency anemia (Brunette, Tran, Wobken, Carlson, & Georgieff, 2010; Fretham et al., 2012; Jorgenson, Wobken, & Georgieff, 2003; Schmidt, Waldow, Grove, Salinas, & Georgieff, 2007). The epigenetic theory posits that environmental factors, such as nutritional deficits, can alter the regulation of synaptic plasticity genes, both during the period of deficiency and into adulthood, thereby altering the function of the adult brain. Neonatal iron deficiency anemia has been shown to induce such effects on targeted genes (e.g., BDNF) and also on networks of genes that are associated with psychopathologies such as schizophrenia and autism (Tran, Kennedy, Lien, Simmons, & Georgieff, 2014; Tran et al., 2016). The effects of such changes in the adult hippocampus have been linked to abnormalities in NOR performance following early‐life ID anemia (Kennedy et al., 2014).

Our data reveal that PIA differentially affects neurobehavioral outcomes in males and females. Although a similar conclusion has been suggested in clinical reports, it has been difficult to disentangle the effects of anemia from other confounds, including its treatment with red blood cell transfusions, which are thought to be pro‐inflammatory. While the mechanism driving these differences has yet to be uncovered, it has been proposed that female preterm infants show greater sensitivity to pro‐inflammatory events (Nopoulos et al., 2011 (though we note that the results of this study should be considered with caution due to poor follow‐up rates); Benavides et al., 2019). Indeed, the finding that females with higher Hct levels due to more numerous red cell transfusions harbored the most significant structural brain abnormalities relative to all other groups in this study seems to support this interpretation (but see a note of caution above). However, anemia itself also has a pro‐inflammatory component, as demonstrated in preclinical models (Arthur et al., 2019). It is thus possible that anemia alone is sufficient to produce a strong pro‐inflammatory response in female pups and that such a response would be commensurate with anemia severity. Since tissue cytokines were not measured in the current set of experiments, the interrogation of the putative relationship between anemia status and inflammatory response remains an objective of future work.

## 5 CONCLUSION

Preterm infants are at increased risk for cognitive, affective, and social deficits. A common practice in NICUs is to order multiple phlebotomies for laboratory testing, resulting in neonatal anemia. Yet, the impact of early‐life anemia on neurodevelopmental outcomes has not been investigated thoroughly. This gap in our knowledge can be partially mitigated through the use of preclinical models. Here, we demonstrate that neonatal PIA in mice results in multiple, sex‐specific behavioral abnormalities that persist into adulthood. These findings have potential translational utility in that they may inform prospective efforts to reduce PIA in preterm infants to secure better neurodevelopmental outcomes in this high‐risk group.

## 6 CONFLICT OF INTEREST STATEMENT

The authors declare no conflicts of interest.

## AUTHOR CONTRIBUTIONS

TMM conducted behavioral experiments and data analyses and wrote the manuscript. GS performed phlebotomies, maintained the breeding colonies, and edited the manuscript. TAG, JCG, and MKG provided revisions to the manuscript, guided experimental design, and assisted with edits of the text and graphs.

### PEER REVIEW

The peer review history for this article is available at https://publons.com/publon/10.1002/brb3.1780.

## Data Availability

All data are available upon reasonable request.
